# Spatial variation and associated factors of deworming among children aged 24 to 59 months in Ethiopia: spatial and multilevel logistic analysis

**DOI:** 10.1186/s12889-022-13156-2

**Published:** 2022-04-09

**Authors:** Abiyu Abadi Tareke

**Affiliations:** Department of Monitoring and Evaluation, West Gondar Zonal Health Department, Gondar, Ethiopia

**Keywords:** EDHS, 2016, Deworming, Spatial analysis, Multilevel logistic regression

## Abstract

**Background:**

Intestinal parasitic infection is one of the neglected tropical diseases (NTD) which is mainly concentrated in developing countries. Gastrointestinal parasitic infections are diseases of poverty, which mainly affects children living in tropical and subtropical regions like Ethiopia. Deworming to children aged 24–59 months of age is one of the strategic initiatives to halt the global burden of intestinal parasitosis among under-five children. The information generated at local levels like hotspot areas (clusters with a high proportion of poor deworming uptake) that were identified in this study could help decision-makers to develop location-based interventional strategies.

**Objective:**

This study was aimed to assess the spatial variation and factors associated with poor deworming uptake among children aged 24–59 months in Ethiopia using evidence from the 2016 Ethiopian Demographic Health Survey (EDHS).

**Methods:**

To explore, create, visualize and edit the spatial information of poor uptake of deworming medication, ArcGIS version 10.8 software was used. The spatial pattern of poor deworming uptake was determined using global spatial autocorrelation. Purely spatial scan statistic was used to identify statistically significant hotspot areas using SaTScan™ version 9.7 software. Multilevel logistic regression analysis was fitted to identify factors associated with poor deworming uptake in Ethiopia. Variables with a *p*-value< 0.2 in the bivariable regression were considered for multivariable regression analysis. Adjusted odds ratio with a 95% confidence interval (CI) and *p*-value < 0.05 were used to declare the statistical significance of each factor.

**Results:**

The spatial pattern of poor deworming uptake in Ethiopia was non-random, i.e. clustered. The most likely cluster was found concentrated in most parts of Somali and East Oromia. Variables like being born from an uneducated mother ((Adjusted Odds Ratio (AOR) = 1.65; 95% CI: 1.16–2.36)), being born from an unemployed mother (AOR = 1. 1.43; 95% CI: 1.19–1.74), being delivered at home (AOR = 1.60 95% CI: 1.27, 2.02), diarrhea in the last 2 weeks (AOR = 0.68, 95%CI: 0.51, 0.90), and region of residency were the significant variables associated with poor deworming medication uptake among children aged to 24 to 59 months in Ethiopia.

**Conclusion:**

The spatial pattern of poor deworming uptake was non-random in Ethiopia. Variables like educational status, employment, distance, place of delivery, diarrhea and region of living were found associated with poor deworming uptake. Tailoring interventional programs based on identified clusters is recommended to minimize this unfavorable deworming uptake.

## Background

Intestinal parasitic infections are one of the neglected tropical diseases (NTD) which is mainly concentrated in developing countries [1]. Soil-transmitted helminths like *roundworms, whipworms and hookworms* are among the most familiar infections to human beings and which found mainly in areas with warm and moist climates where sanitation and hygiene are poor [2, 3]. Those soil-transmitted helminths occupy the intestine of human beings and their eggs are passed in the feces of infected persons [3]. If an infected person defecates outside (near bushes, in a garden, or field) or if the faeces of an infected person are used as fertilizer, eggs are deposited on the soil [3]. Among the most common cause of intestinal parasitic infections are *worms, Enterobius vermicularis, Amebiasis trichuriasis, Giardia lamblia, Ancylostoma duodenale, Necator americanus, and Entamoeba histolytica* are the most common species [4, 5]. Gastrointestinal parasitic infections are diseases of poverty, which mainly affects children living in tropical and subtropical regions like Ethiopia [6]. Worldwide, more than one billion people are influenced by gastrointestinal parasitic infections and majority of them are children [7]. Children under the age of 5 years are more prone to intestinal parasitosis because of their delicate immunity system and mouthing of contaminated soil or object [8]. Those infections are more common in countries with low level of Water, Sanitation and Hygiene (WASH). In Ethiopia, the availability of WASH services are poor. For example, research done using the 2016 Ethiopian demographic health survey’s datasets revealed that the proportion of households having access to improved drinking water sources and toilet facilities were relatively low(i.e.70% for drinking water and 25.4% for toilet facilities) [9]. Another pocket study done in the Tigray region of Ethiopia also showed a similar occasion (i.e. Poor quality of WASH in the country) that revealed that only 53, 42.4 and 36.2% households utilize latrine, hand washing, and water facilities properly [10].

Additionally, the prevalence of gastrointestinal parasitic infection is still high in Ethiopia. A systematic review in Ethiopia showed that the pooled prevalence of intestinal parasitosis was 48%. Intestinal parasitic infection can lead to under-five mortality by inducing bloody diarrhea, chronic loss of blood that ends up with anemia, impair intestinal food absorption, and loss of appetite [11, 12]. The influence of helminthic infections goes beyond these obvious health effects to include economic and social effects resulting from lost school attendance and effective work time. Deworming to children aged 24–59 months of age is one of the strategic initiatives to halt the global burden of intestinal parasitosis among under-five children. Deworming (sometimes called Preventive chemotherapy) is the administration of albendazole or mebendazole drug to a high-risk group of population to treat soil-transmitted helminthiases (STH) and is also administrated to minimize future parasitic related morbidity [13]. Besides, a drug like praziquantel is used to treat schistosomiasis [14]. World Health Organization (WHO) recommended annual single-dose administration of either Albendazole (400 mg)or Mebendazole (500 mg) to regions that have a baseline prevalence of any soil-transmitted infection of 20% and Biannual if the baseline prevalence raised above 50% [2]. Despite Ethiopia is launching deworming programs to intervene against the burden of intestinal parasitic infection among preschool-age children and the coverage of preventive chemotherapy (deworming) is still not satisfactory.

Existing studies are believed to embody different limitations, which this paper is designed to address. Firstly, prior research in Ethiopia was conducted by the standard binary logistic regression to find factors associated with deworming uptake among children aged 24–59 months. Ordinary/standard binary logistic regression doesn’t account for the hierarchical nature of Demographic Health Survey (DHS) data, and this might have biased the model estimation. But this study applied multivariable multilevel logistic analysis for such kind of hierarchical data to increase the statistical power and to get the appropriate estimation. Secondly, previous studies in Ethiopia didn’t take into consideration the spatial pattern of deworming uptake among children aged 24–59 months. Sub-regional estimates of poor deworming uptake using spatial modeling could give information to local decision-makers about what is happening at local (geographic) and administrative levels. The information generated at local levels like hotspot areas (clusters with a high proportion of poor deworming uptake) could help decision-makers to develop location-based interventional strategies. Therefore, this study was aimed to assess the spatial variation and factors associated with poor deworming uptake among children aged 24–59 months in Ethiopia using evidence from the 2016 Ethiopian Demographic Health Survey (EDHS).

## Methods

### Study area, data source and study period

This study utilized a dataset of the Ethiopian fourth demographic health survey which was conducted from January 18, 2016, to June 27, 2016. The first administrative level of the country is composed of nine Regional States: Tigray, Afar, Amhara, Oromia, and Somali, Southern Nation Nationalities and Peoples Region (SNNPR), Benishangul-Gumuz, Gambela, and Harari; and two City Administrations council of Dire Dawa and Addis Ababa. Every first- level administration are divided into Woredas (districts) and Kebele (sub-districts) [15].

### Sampling technique and study population

The 2016 EDHS utilized Enumeration areas of the Population and Housing Census (PHC) as a sampling frame. The 2016 EDHS sampling design was a multistage stratified sampling strategy. The sampling procedure was two-staged ways of selecting the study sample. In the first stage of selection, 645 primary sampling units (PSU) (202 in urban areas and 443 in rural areas) or EAs (enumeration areas) were from the 11 first administrative levels based on the proportion of EAs they contributed. In the second stage of selection, a fixed number of 28 households per each EAs were selected, yielding a total of 15,683 women were eligible for interview. Finally, total weighted sample of 5949 children aged 24–59 months were embodied in this study.

### Study variables

The dependent variable was deworming status, which was dichotomized as “poor” and “good”. Poor deworming uptake (i.e. a child who had not taken deworming medication), which was labeled as “poor” and coded 1. A child who has taken supplementary deworming medication was said good with deworming drug and labeled as “good” and coded 0.

### Data management and analyses

Cross tabulations and summary statistics were done using STATA version 16. The forest plot technique was utilized to display the magnitude of poor deworming medication uptake across the administrative regions. To plot the 95% confidence interval (CI) of the coefficient of each variable of the best-fitted model, the STATA command “coefpot” was applied. We applied survey commands to consider the complex nature of the DHS design and to restore representativeness.

### Spatial analyses

To explore, create, visualize and edit the spatial information of poor uptake of deworming medication, ArcGIS version 10.8 software was used. To declare whether the spatial configuration of poor deworming uptake was scattered, clustered, or uniformly distributed across the study area using the global spatial autocorrelation model, Moran’s I value was calculated [16]. Moran’s I value close to − 1 indicates that poor deworming medication uptake is spatially dispersed or unrelated, whereas the closer the Moran’s I value to a positive one demonstrates poor deworming drug uptake are spatially related (clustered). But, if the Moran’s I value is more proximal to 0 it denotes that random distribution. Besides, a statistically significant (*p*-value < 0.05) and Moran’s I result with a positive z-score value indicates that clustering of deworming drug uptake, while a negative z-score value indicates spatial dispersion of deworming drug uptake. Moreover, the ordinary Kriging method of spatial interpolation was done to predict the proportion of poor deworming uptake among children aged 24–59 months in unmeasured locations by forming weights from surrounding measured values.

To quantify and explore spatial dependency (spatial autocorrelation) of poor deworming uptake, the Semivariograph model was used. In addition to this, Getis-Ord Gi* statistics were computed to measure how spatial autocorrelation varies over the study location by calculating the GI* statistic for each area. Z-score and *p*-value were calculated to declare the statistical significance. Statistical output with high GI* indicates a “hotspot” area, whereas, low GI* means a “cold spot”. Statistical significance of clustering was certified at *p*-value of less than 0.05 and 95% CI. If the *p*-value < 0.05 and the z-score is less than − 1.96, it was declared as cold spot and if Z-score is greater than + 1.96 it was declared as a hotspot areas [17].

Purely spatial scan statistic was used to identify statistically significant hotspot areas using SaTScan™ version 9.7 software. This purely spatial scan statistic was the Bernoulli model- based with 1/0 event data, such as cases (1) (respond “no” to uptake of deworming medication) and controls (0) (respond “yes” to uptake of deworming medication). In this dataset, the controls are calculated by subtracting the cases of each cluster from total number of 24–59 months of children of the respective cluster. To allow maximum-sized clusters, the default setting (50% of the population at risk) was used. While running spatial analysis, 21 clusters with missing coordinates and 5 clusters with zero observations were excluded from the study.

### Mixed model

Both bivariable and multivariable multilevel logistic regression models were fitted to identify factors associated with poor deworming uptake in Ethiopia. Multilevel analysis is useful for nested data like DHS. For instance, there are individual-level characteristics, such as each mother’s education and her household wealth status. We expect that, the greater the mother’s income and education, the higher her uptake of deworming medication for her children aged 24–59 months. But, the mother’s level characteristics i.e. income and Education might be predicted by enumeration area-level/community level characteristics like place of residency, community level media exposure, community-level poverty or regions in which they reside. For example, mothers from wealthy community might have higher enthusiasm to bring here child to health facility for deworming. In DHS data, the scores of individual level characteristics of children are more likely to be more correlated within cluster/ enumeration area than with the scores from another clusters. Similarity of score of individual characteristics within cluster might consequence violation of observation independency assumptions of classical regressions. Multilevel analysis can address the lack of independence of the observations while analyzing nested/hierarchical data like EDHS.

Two-level binary logistic regression (i.e. individual and community level) was fitted to identify factors that associated with poor deworming medication uptake. Four models were fitted. Of the four models, null model (model not including independent variables) also called random intercepts model was fitted to calculate the extent of cluster variability on poor uptake of deworming medications. Model fitness was assessed using different fitness parameters like the Likelihood Ratio test (LR), deviance, Akaike Information Criteria (AIC) and Bayesian Information Criteria (BIC). The model with the lowest of the four fitness parameters was selected as best fitted model. Cluster variability was assessed through calculating Intra-Class Coefficient (ICC), median odds ratio (MOR) and Proportional Change in Variance (PCV). ICC measures the percentage variation attributed by the community-level variables and while PCV measures the proportional change in the community-level variance between the null model and the succeeding models [18]. The MOR describes the area-level variance as an Odds Ratio (OR), as the median value of the distribution of ORs obtained when two children with the same covariate values are picked from two different areas, comparing the one from the higher poor deworming uptake with the one from the area with lower poor uptake of deworming medication. In the absence of any area-level variation, the MOR is equal to 1. The value of ICCs and MORs was estimated from intercept-only models (null model) to examine the presence of clustering and heterogeneity between areas in the outcomes of poor deworming medication uptake. Finally, Variables with *p*-value< 0.2 were considered for multivariable analysis. Adjusted odds ratio with 95% CI and *p*-value < 0.05 were used to declare statistical significances of factors.

### Ethical consideration

This study used a dataset of national representative demographic health surveys. Therefore, ethical approval is not required. But, datasets utilized for this study were requested by providing a clear explanation of the objectives and necessity of this study. I registered and requested the DHS dataset to the online database (www.dhsprogram.com) and received an authorization letter to download the requested dataset.

## Results

A total of 5609 children aged 24 to 59 months was included in this study. The median age of the study participants was 41 months ((Inter Quartile Range (IQR) = 32, 50)). The majority of the study participants were born to uneducated mothers (69.4%) and were males (86%). More than three-fourths of the children were born at health facilities (Table 1).

**Table 1:** Characteristics of the study participants in Ethiopia, 2016 (*n* = 5609)

Characteristics	Weighted frequency	Percent
**Maternal age**		
15–24 years	1028	17.28%
25–34 years	3267	54.90%
35–49 years	1655	27.82%
**Maternal education**		
None	4127	69.37%
Primary school	1458	24.51%
Secondary and above	364	6.12%
**Working status**	3176	53.40%
Working	2773	46.60%
not working		
**Sex of household head**		
Male	5111	85.90%
Female	837	14.00%
**Family Wealth status**		
Poor	2882	48.45%
Middle	1201	20.20%
Rich	1865	31.36%
**Perceived distance to health facilities**		
Big problem	3624	61.00%
Not big problem	2324	39.00%
**Health related decision making autonomy**		
Husband only	1239	21.95%
Jointly	3683	65.24%
Mother alone	723	12.80%
**Place of delivery to the last birth**		
Home	4754	79.92%
Health facility	1194	20.08%
**Sex of the child**		
Male	3218	54.10%
Female	2730	45.90%
**Age of the child(months)**		
24–35 months	1830	31.80%
36–47 months	1903	33.00%
48–59 months	2028	35.20%
**Diarrhea in the last 2 weeks**		
No	5423	91.16%
Yes	526	8.84%
**Place of residency**		
Urban	625	10.50%
Rural	5323	89.50%
**Region**		
Tigray	360	6.06%
Afar	63	1.06%
Amhara	1125	18.92%
Oromia	2574	43.26%
Somali	291	4.88%
Benishangul	69	1.16%
SNNP	1291	1.16%
Gambela	15	21.71%
Harari	14	0.23%
Addis Ababa	121	2.04%
Dire Dawa	25	0.43%
**Community poverty**		
Low	3659	61.50%
High	2290	38.50%
**Community literacy**		
Low	3044	51.20%
High	2905	48.80%

**Fig. 1:**
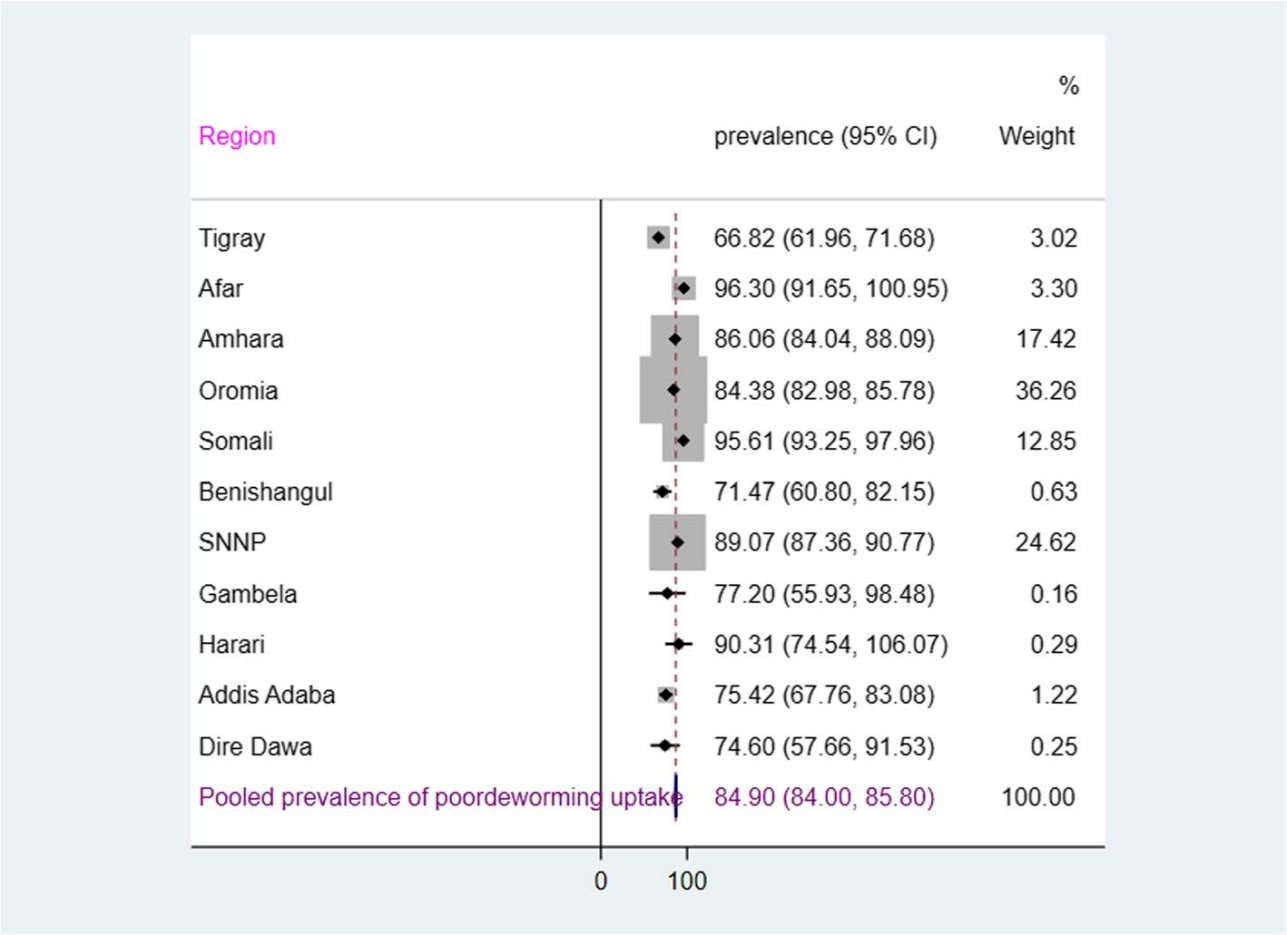
Forest plot of prevalence of deworming medication uptake among children of 24–59 months in Ethiopia, 2016

The overall prevalence of poor deworming uptake in Ethiopia was 85% [95% CI: 83.0–86.8]. Regional variation of poor deworming uptake was observed in Ethiopia. Region like Afar (96.3%), Somali (95.6%), Harari (90.3%), SNNP (24.6%) and Gambela (89%) and Amhara (86%) had unfavorable (high) proportion of poor deworming medication uptake relative to pooled national prevalence i.e. 85%. However, a relatively lower proportion of poor deworming medication uptake among children aged 24–59 months were noted in Tigray (66.82%), Benishangul (71.5%) and Dire Dawa (74.6%) provinces (Fig. 1).

### Spatial distribution of poor uptake of deworming

**Fig. 2:**
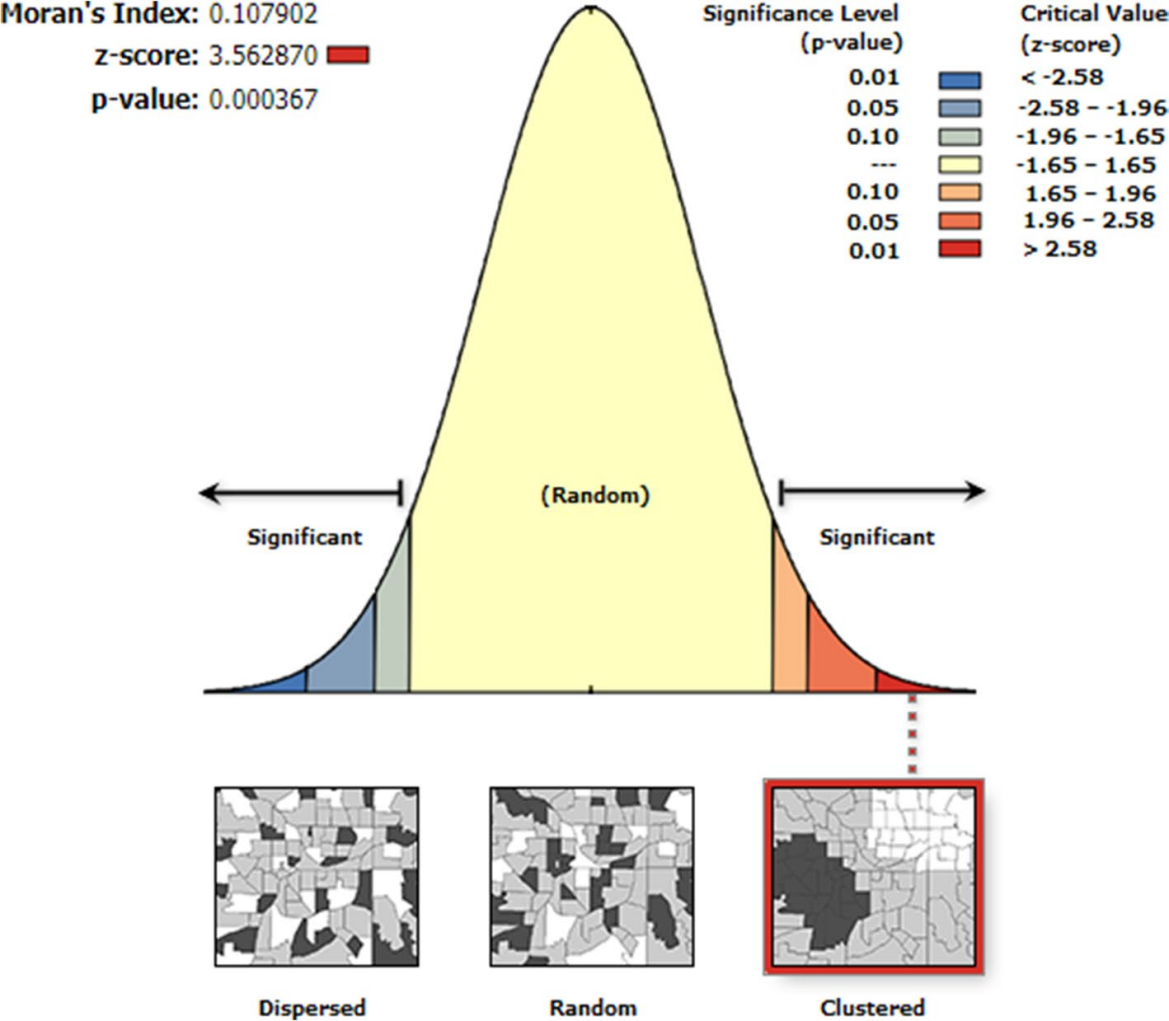
Spatial pattern of poor deworming drug up in Ethiopia, 2016

The Moran’s index (Moran’s I) value of 0.1079 with a statistically significant *p*-value and positive z-score suggests that poor deworming medication among 24–59 months children was not randomly distributed i.e. clustered (Fig. 2).

### Hotspot and cold spot analysis of poor deworming uptake

**Fig. 3:**
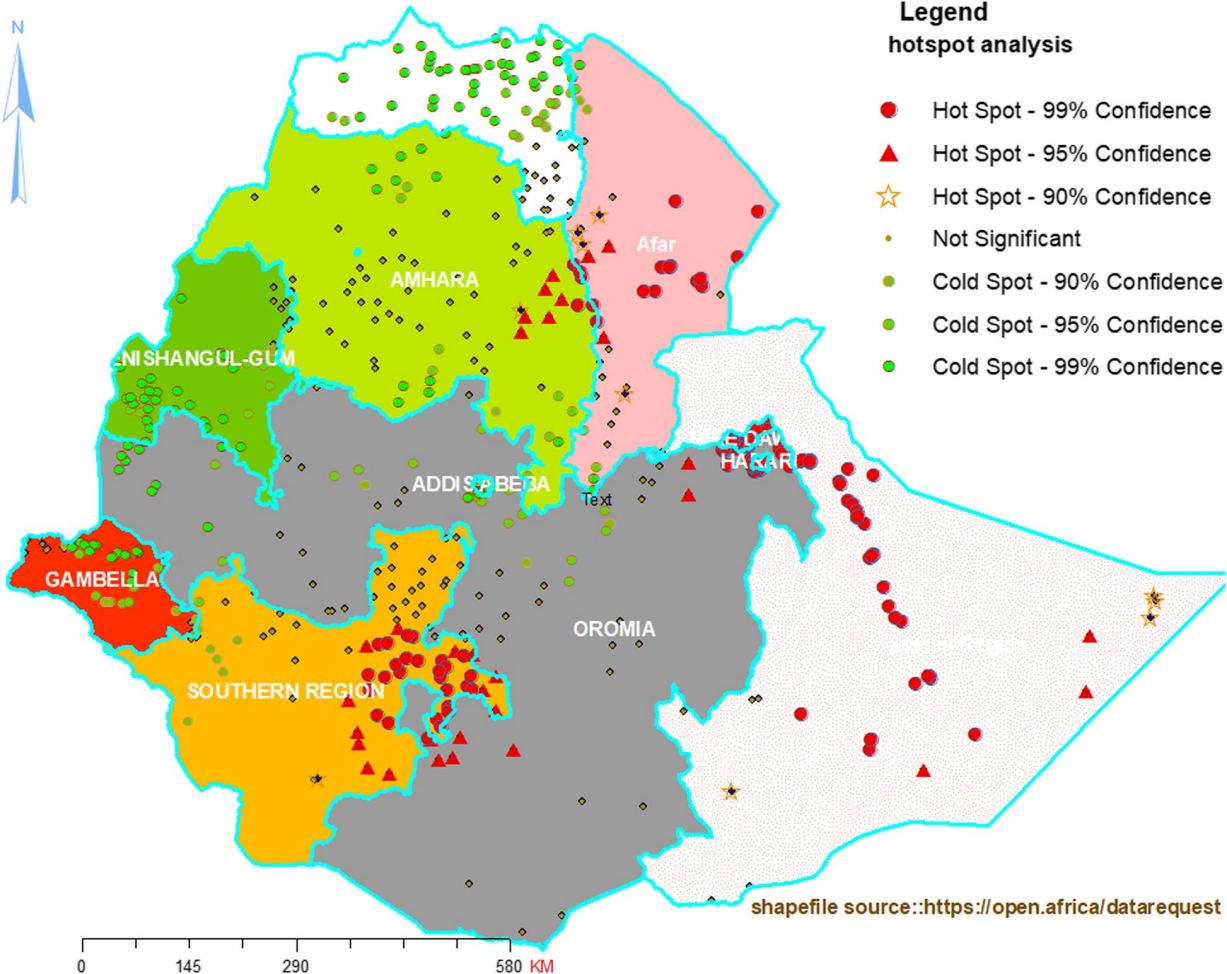
Hotspot & cold spot sites of poor deworming in children 24–59 months in Ethiopia, 2016

The red color indicates areas with the highest proportion of poor deworming uptake (hot spots), which was concentrated in most of the Somali and Afar regions, some parts of East Amhara, most of East SNNP and Harari. In opposite, the green color represents a lower statistical significant proportion of poor deworming uptake (cold spot), which was located in most areas of Tigray region, southeast and north part of Amhara region, most area of Gambela, south Benishangul and west and central part of Oromia region (Fig. 3).

### SaTScan analysis for not taking deworming medication

**Fig. 4:**
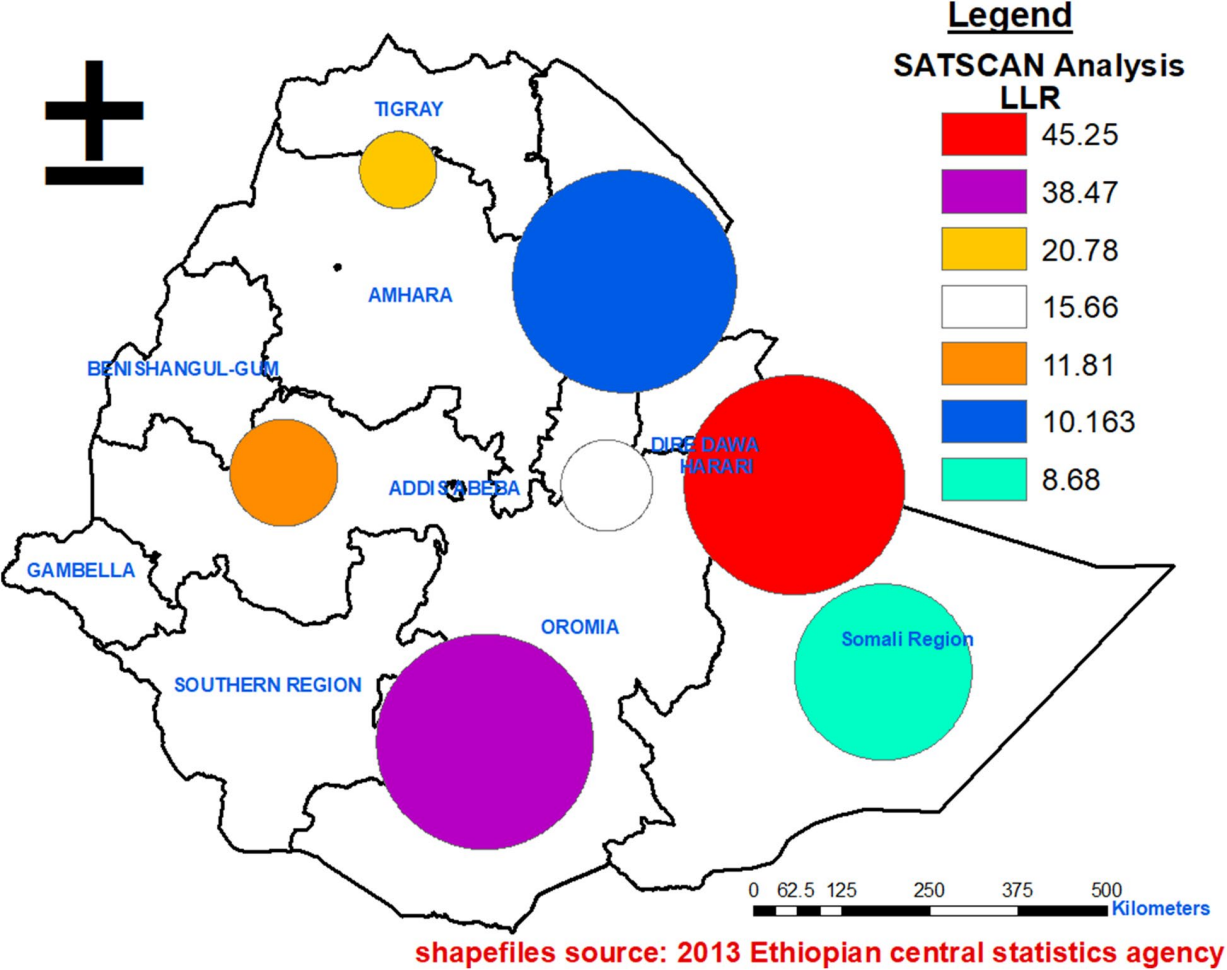
Spatial sat scan analysis of the proportion of children with poor deworming drug in Ethiopia, 2016

From Kulldorff’s scan statistic, the Percent cases in the area (prevalence of poor deworming drug uptake) were 85% (Table 2). The Kulldorff’s spatial scan analysis detected seven clusters (statistically significant high-risk areas to poor deworming uptake). All the seven detected clusters had poor uptake of deworming medication than expected. The primary cluster ((log likelihood ratio (LLR) = 45.25, RR = 1.17, *p*-value < 0.0001)) is also called the most likely cluster, i.e. the cluster less likely to have occurred by chance was found concentrated to a great extent of north Somali and south part of East Afar regions. The center of the primary cluster area was located at (9.107168 N, 43.165843 E) / 155.70 km radius. The relative risk of 1.17 indicates that children inside the scan window are 17% riskier of encountering poor deworming drug uptake compared to children outside the window (Table 3 and Fig. 4).

**Table 2:** SaTScan Model summary

Types of Probability model used	Bernoulli model
Scan area with	High rates
Number of locations (v001)	617
Total population (total 24–59 months children)	5792
Total number of cases (poor deworming drug uptake)	4921
Percent cases in area (prevalence of poor deworming drug uptake)	85%

**Table 3:** Statistically significant cluster of poor deworming drug uptake among children aged 24–59 months in Ethiopia

Types of cluster	Coordinates/Radius	N	Cluster location	Expected cases	Observed cases	RR	LLR	*p*-value
Most likely cluster	(9.107168 N, 43.165843 E) / 155.70 km	105	North Somalia and south Afar	320	371	1.17	45.25	< 0.0001
Secondary cluster 1	(5.844300 N, 39.182881 E) / 153.22 km	25	South Oromia and East SNNP	553	620	1.14	38.47	< 0.0001
Secondary cluster 2	(13.159408 N, 38.054771 E) / 54.65 km	5	North Amhara	107	126	1.18	20.78	< 0.0001
Secondary cluster 3	(9.120627 N, 40.753382 E) / 64.80 km	8	North Oromia near Harari region	196	222	1.14	15.66	< 0.001
Secondary cluster 4	(9.278782 N, 36.595749 E) / 76.19 km	4	North Oromia near the border with Benishangul region	61	72	1.18	11.83	< 0.01
Secondary cluster 5	(11.726886 N, 40.997480 E) / 158.12 km	34	Central Afar, East Amhara and north Somalia regions	53	62	1.18	10.16	< 0.05
Secondary cluster 6	(6.708449 N, 44.273542 E) / 124.84 km	11	Central Somalia region	45	53	1.18	8.68	< 0.05

N = total numbers of enumeration areas incorporated in the respected cluster

The remaining lists of secondary clusters were located between the border of the east part of SNNP and the west part of Oromia (secondary cluster 1), in between the boundary of south of Tigray and north of Amhara regions (secondary cluster 2), at the boundary of north of Oromia region and southern portion of Afar region (secondary cluster 3). Secondary cluster 4 mainly covers, northern portions of Oromia region. Secondary cluster 4 covers almost all southern portions of Afar and Eastern portions of Amhara region (Table 3 and Fig. 4).

### Interpolation of poor deworming medication uptake

**Fig. 5:**
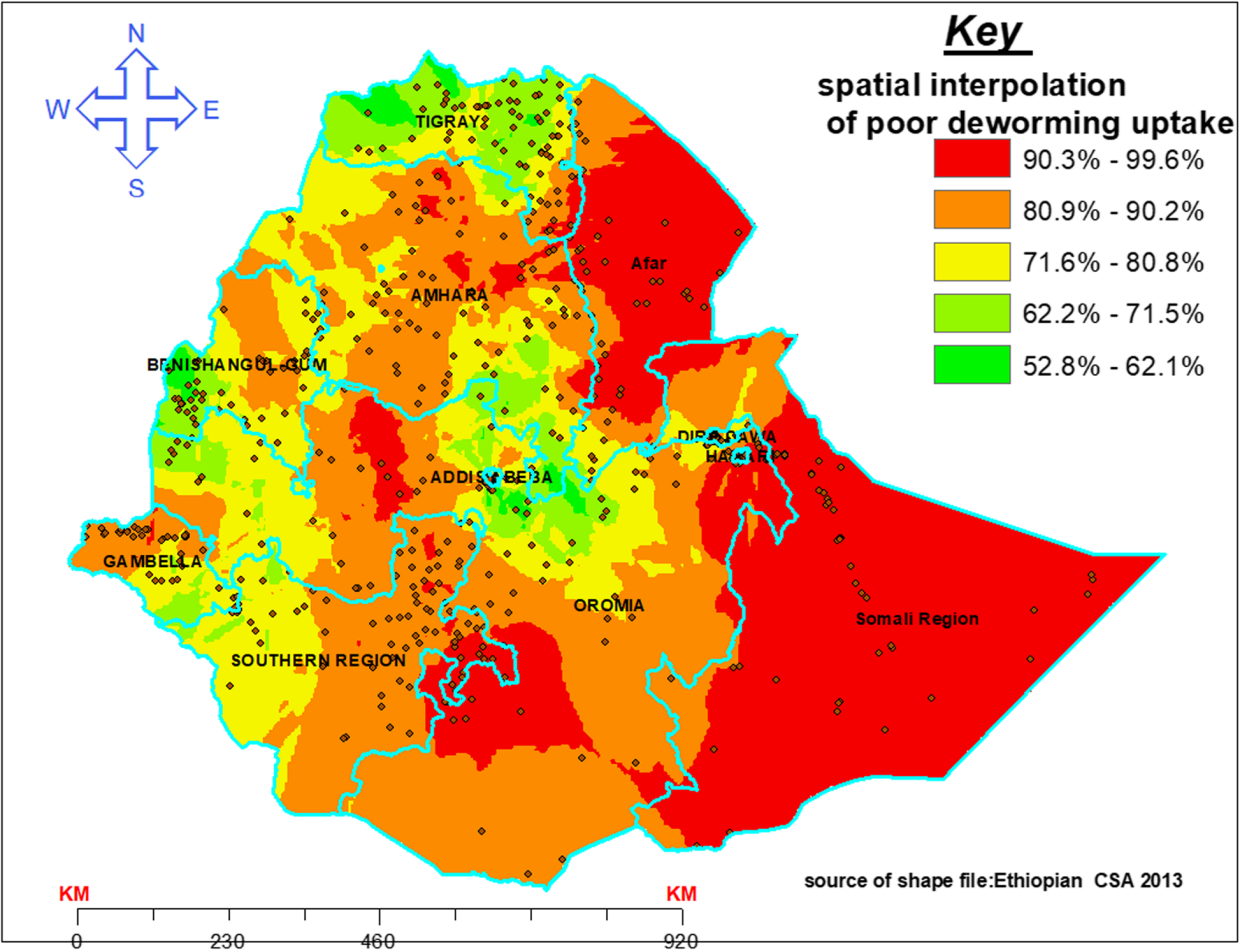
Kriging spatial interpolation of poor deworming drug uptake in Ethiopia, 2016

From the Kriging spatial interpolation, a high proportion of predicted poor deworming drug uptake (i.e. the red shades) covers most parts of Afar and Somali regions, some parts of (south, East and central Oromia) and some parts of (north, east and central Amhara). On the contrary, favorable proportion of uptake of deworming drug (the green shade) was observed in most parts of Tigray, western Benishangul, central Oromia, Addis Ababa and some southern portions of the Gambela region (Fig. 5).

### Factors associated with poor uptake of deworming

#### Model comparison

The ICC provides a measure of the proportion of the variance in the outcome between the level-2 Units. In this study where children (the level-1 units) are grouped within clusters (the level-2 units), an ICC of 0.33 from the null model would mean that 33% of the variability in the odds of poor uptake of deworming medication was explained by community-level factors and the remaining 67% of the variation was due to differences between children. Furthermore, median odds ratio (MOR) of 3.35 conveys that, the median value of OR between clusters at high risk of poor deworming uptake and clusters at lowest risk of poor deworming uptake when randomly choosing two children having the same individual-level characteristics but from a different cluster, the increased risk of poor deworming uptake when shifting from low risk to high risk is 3.35 times. The value of MOR and ICC indicates a justifiable reason for conducting multilevel logistic analysis (Table 4).

**Table 4:** Model comparison and fitness parameter outputs

Fitness parameter	Null Model	Model I	Model II	Model III
Community level variance	1.62 [95% CI: 1.27, 2.08]	1.2 [95% CI: 0.90, 1.60]	0.83 [95% CI: 0.61,1.12]	0.76 [95% CI: 0.55,1.07]
Community level variance(se)	0.2043788	0.1738411	0.1282981	0.1310076
ICC	33%	26.8%	20%	18.8%
MOR	3.35 [95% CI: 2.92, 3.94]	2.83 [95% CI: 2.46, 3.32]	2.38 [95% CI: 2.10, 2.73]	2.3 [95% CI: 2.0, 2.7]
PCV (%)	baseline	25.9%	48.8%	53%
**Model fitness**				
Log- likelihood ratio(LLR)	− 2332	− 2069.6	2236.5	−2006.5
DIC(−2LLR)	4664	4139.2	4473	4013
AIC	4668	4183	4503	4082.9
BIC	4681	4327	4603	4312.8

Proportional Change in Variance (PCV) or Change in Community-level Variance value of the full model (model III) indicates that about 53% of the variance in the odds of poor deworming uptake across the community/ class was attributed to the effect of both level − 1 and level-2 factors.

From the above table, we can understand that the full model (model IV) i.e. model containing individual and community level factors is the best-fitted model with this data. This is because Model IV has a low value of deviance, BIC and AICc.

**Fig. 6:**
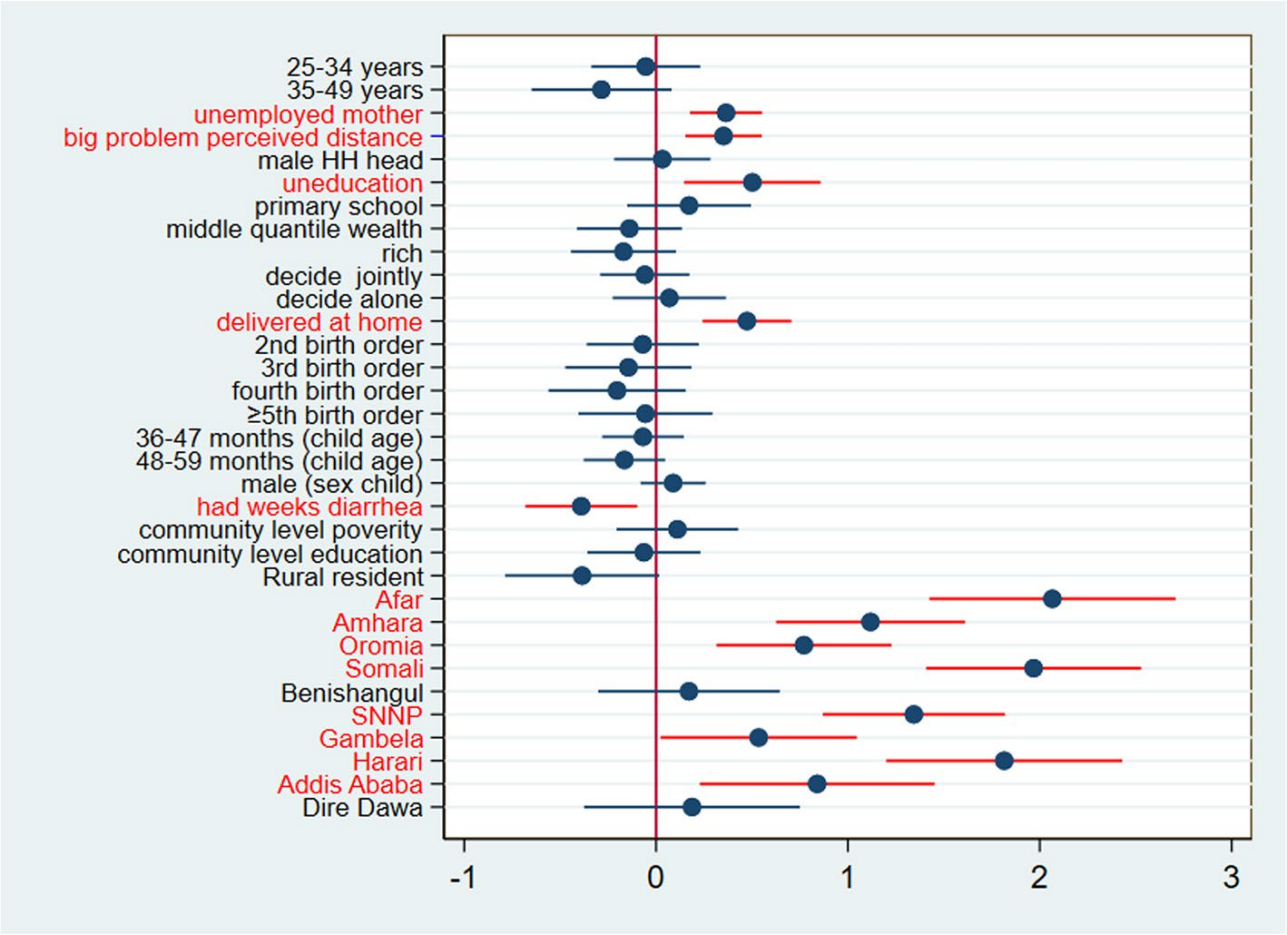
Coefficient plot of community and individual-level factors (model IV) of poor uptake of deworming among children aged 24–59 months in Ethiopia, 2016

From the below coefficient plot of explanatory variables of poor deworming uptake, the red line represents 95% CI of the coefficient of the respective independent variable and the dot at the middle of the line represents the coefficient point estimate. Variables with the line that crossed the vertical line were considered as insignificant variables and these which didn’t touch or cross the vertical line are considered statistically significant variable. Variables with a horizontal line that didn’t cross the vertical and lay to the left of the vertical line were variables which negatively associated with poor uptake of deworming medication, and to the right of the vertical line were variables positively associated with the outcome variable (Fig. 6).

Therefore, variables like children whose mothers were unemployed, perceived distance to a nearby health facility as a big problem, being uneducated mothers, being delivered at home and region of living (all except Benishangul and Dire Dawa) were positively and significantly associated with poor uptake of deworming medication. Conversely, diarrhea in the last 2 weeks was the only negatively associated variable with poor deworming medication uptake (Fig. 6 and Table 5).

**Table 5:** Multivariable multilevel analysis result of poor deworming among children of 24–59 months in Ethiopia

Characteristics	null model (95%CI AOR)	Model I (95%CI AOR)	Model II (95%CI AOR)	Model III (95%CI AOR)
Age group				
15–24		Ref		Ref
25–34		0.95 [0.72, 1.26]		0.95 [0.71, 1.26]
35–49		0.73 [0.51, 1.04]		0.75 [0.52, 1.08]
**Educational status**				
Secondary and above		Ref		Ref
None		1.70 [1.20, 2.40]		1.65 [1.16, 2.36]***
Primary		1.14 [0.82, 1.57]		1.19 [0.86, 1.64]
**Working status**				
working		Ref		Ref
Not working		1.73 [1.43, 2.08]		1.43 [1.19, 1.74]**
**Sex of household head**				
Female		Ref		Ref
Male		0.91 [0.71, 1.18]		1.03 [0.80, 1.32]
**Wealth status**				
Poor		**Ref**		**Ref**
Middle		0.80 [0.61, 1.03]		0.87 [0.66, 1.14]
rich		0.86 [0.67, 1.09]		0.84 [0.64, 1.10]
**Perceived distance**				
Not big problem		**Ref**		**Ref**
Big problem		1.31 [1.08, 1.60]		1.42 [1.16, 1.73]***
**Health related decision maker**				
Husband		**Ref**		**Ref**
Jointly		0.92 [0.73, 1.17]		0.94 [0.74, 1.19]
Alone		1.08 [0.80, 1.46]		1.07 [0.80, 1.43]
**Place of delivery**				
Facility		**Ref**		**Ref**
Home		1.72 [1.38, 2.16]		1.60 [1.27, 2.02]***
**Birth order**				
1st		**Ref**		**Ref**
2nd		0.97 [0.72, 1.30]		0.93 [0.70, 1.25]
3rd		0.86 [0.62, 1.20]		0.87 [0.62, 1.20]
4th		0.82 [0.57, 1.17]		0.82 [0.57, 1.17]
≥ 5th		0.96 [0.68, 1.37]		0.95 [0.67, 1.34]
**Sex of child**				
Male		**Ref**		**Ref**
Female		1.07 [0.91, 1.30]		1.09 [0.92, 1.30]
**age of child**				
24–35		**Ref**		**Ref**
36–47		0.92 [0.74, 1.14]		0.93 [0.76, 1.16]
48–59		0.85 [0.70, 1.05]		0.85 [0.69, 1.05]
**Diarrhea in the last 2 weeks**				
No		**Ref**	**Ref**	**Ref**
Yes		0.68 [0.50, 0.91]		0.68 [0.51, 0.90]**
**Community level factors**				
**Place of residency**				
Urban			**Ref**	**Ref**
Rural			0.94 [0.66, 1.34]	0.68 [0.46, 1.01]
Region				
Tigray			Ref	**Ref**
Afar			0.08 [0.05, 0.16]	**7.89 [4.15, 15.00]**
Amhara			0.30 [0.19, 0.49]	**3.06 [1.87, 5.00]**
Oromia			0.36 [0.23, 0.56]	**2.16 [1.37, 3.41]**
Somali			0.10 [0.06, 0.17]	**7.16 [4.08, 12.53]**
Benishangul			0.76 [0.48, 1.20]	1.19 [0.74, 1.90]
SNNP			0.22 [0.14, 0.35]	**3.83 [2.40, 6.16]**
Gambela			0.47 [0.29, 0.76]	**1.71 [1.02, 2.85]**
Harari			0.16 [0.09, 0.29]	**6.14 [3.31, 11.36]**
Addis Ababa			0.49 [0.27, 0.87]	**2.32 [1.26, 4.30]**
Dire Dawa			0.60 [0.35, 1.04]	**1.21 [0.70, 2.12]**
**Community poverty**				
Low			**Ref**	**Ref**
High			0.77 [0.60, 1.02]	1.12 [0.81, 1.53]
**Community literacy**				
Low			**Ref**	**Ref**
High			1.37 [1.04, 1.81]	0.94 [0.70, 1.26]

*Note*: * indicates statistical significance at a *p*-value less than 0.05, model I: model containing only dependent variable, Model II: model containing only individual-level factors, Model III: model containing only community-level factors, Model IV: full model containing both individual and community factors. Ref = reference category

The Multivariable multilevel logistic analysis estimates show that the risk of poor deworming medication uptake is slightly higher for children born to mothers who were not educated (AOR = 1.65; 95% CI: 1.16–2.36) compared to having secondary and above education level. The odds of poor uptake of deworming medication was 43% higher in children born to unemployed mothers (AOR = 1. 1.43; 95% CI: 1.19–1.74) relative to employed one. Similarly, the odds of poor uptake of deworming for a child born to a mother who gives birth at home was 60% higher relative to giving birth in a health facility (AOR = 1.60 95% CI: 1.27, 2.02) (Table 5).

Moreover, the odds of poor deworming uptake was 42% higher in a child born to mother who perceived the distance from health facilities as a big problem compared to counterparts(AOR = 1.42, 95%CI: 1.16–1.73). Conversely, the odds of poor medication uptake was 32% lower in a child who had diarrhea in the last 2 weeks before the time of the survey time compared to counterparts (AOR = 0.68, 95%CI: 0.51, 0.90).

Regarding community level factors, region of residency was the only significant variable. In particular, children born to mothers who are living in the region of Afar (AOR = 7.9; 95% CI 4.15–15.00), Somali (AOR = 7.16; 95% CI: 4.08–12.53), Harari (AOR = 6.14; 95% CI -15.03.31–11.36), SNNP), SNNP(AOR = 3.83; 95% CI 2.40–6.16) and Amhara (AOR = 3.06; 95% CI: 1.87–5.00) had the highest risk of poor medication uptake compared with those living in the Tigray region.

## Discussion

The overall magnitude of poor deworming uptake among children aged 24–59 months was high (i.e. 85%). The spatial pattern of poor deworming uptake among children in Ethiopia is not random (clustered). Employment status, perceived distance, mother’s education, place of delivery, region of residency and diarrhea were the main factors associated with deworming uptake among children. The spatial analysis identified five clusters of poor deworming uptake in eastern Ethiopia.

The most likely cluster occurred in the Somali regional state (area: 153.22 km, RR: 1.17 and *p*-value< 0.0001) (Table 3). This high proportion of poor deworming uptake in Somali and Afar region might be related to low maternal and child service utilization rate [19]. There were also documented higher perceived health care access challenges in Somali and Afar regions [20]. Those two regions are also among the regions with the lowest Human Development Index [21]. Besides, the observed cluster of poor deworming uptake in Somali region might be explained by poor health-seeking behavior toward child health [22]. Relative to other regions, Gambela, Addis Ababa and Benishangul gumuz region have good deworming medication uptake (i.e. no clustering of cases was observed there) [23]. This might be due to good maternal health uptake in those areas. Having good maternal health services uptake might lay the foundation to take deworming medication for their children. Focused interventions in these high-burden clusters (high proportion of poor deworming uptake areas) can optimize resources and achieve the WHO targeted soil-transmitted helminthiases coverage, i.e. reaching up to 75% of preschool children in need of treatment [24].

The spatial Kriging interpolation assured that a higher proportion of predicted poor uptake of deworming was found, concentrated mainly in Afar and Somali regions (Fig. 5). This can be explained by the pastoralist’s nature of the people in those areas might lead theme to confront difficulty to access health facilities because of no permanent residency [25]. Another reason for this finding might be related to the shortage of trained health professionals in those areas [22]. In Somali, prior research documented that husbands were reported as the predominant decision-maker with regard to children’s health [26]. This restricted autonomy of power over their child’s health has been reported as a major barrier to women’s ability to access child health care services [27, 28]. The interpolated poor deworming uptake could help decision-makers and local authorities in evaluating the performance of prior installed deworming programs, allocate resources and develop targeted intervention plans without conducting additional research.

The multivariable multilevel logistic analysis revealed that employment status, perceived distance, mother’s education, place of delivery and diarrhea were the individual-level factors associated with poor deworming uptake. In addition to this, the region of residency was the only significant variable among the community-level factors. This study showed that children born to uneducated mothers are more likely not to take the deworming drugs. This result is in agreement with another study [29]. Another research in Pakistan [30] also support this finding, which found that the odds of child health care practice were lower among children born to not educated mother. This might be explained by; educational status is more likely to correlate with wealth status, which leads to lack of money to afford cost of transportation and cost of health services.

The current study revealed that regardless of other variables, being a child born to not working/unemployed mother increases the odds ratio of not deworming to children from working women. This finding is in line with a study conducted in Ghana [29], which disclosed that employed women are more likely to utilize deworming for their children compared to unemployed women. This might be due to not working women are less granted to afford child health services and cost of transformation.

This study revealed that being a child born at home was associated with higher odds of not taking deworming medication. A supportive study in Kenya [31] reached a similar conclusion to this finding. This might be justified by; women who deliver at home are expected to have unfavorable awareness towards the health of their child compared to their counterparts. In addition to this, women who were not given birth at health facilities are less likely to receive advice about their future health of the child specifically advice about the importance of deworming drug supplementation during early childhood.

Furthermore, children who had diarrhea 2 weeks before the survey were less likely to take deworming drugs compared to those who had no diarrhea episode. This might be the relationship between diarrhea and intestinal parasitic infection [32]. Children who had encountered diarrhea are more likely to seek health services, and the diarrhea episode could have created an opportunity to take deworming tablets.

From the community level factors, group regions of residency have increased the odds of not taking deworming drugs. Children residing in regions Afar, Somali, Harari, SNNP and Amhara has found increased odds of not taking deworming tablets. A similar supportive study from Ghana revealed that, residents from the western part of the country were less likely to take deworming tablets [29]. This might be justified by the unequal distribution of health services and other Infrastructures throughout the country.

## Conclusion

To conclude, the distribution of not taking deworming drug among 24–59 months of children was unevenly distributed across space and major public health concern in Ethiopia. Maternal education, perceived distance to health facility, and place of delivery to the last child, maternal working/employment status, region of living and diarrhea in the last 2 weeks prior to date of interview were statistically significant factors associated with unfavorable deworming drug uptake in Ethiopia. Developing targeted interventional programs towards the clusters identified by scan statistics are recommended to minimize this unfavorable deworming uptake. Additionally, enhancing mothers through education and employment, and increasing access towards facility delivery are also recommended to halt this public health problem.

## Data Availability

The datasets and other materials are available on the DHS program measure website: www.dhsprogram.com. It is also available from the corresponding author on reasonable requests.

## Abbreviations

AIC: Akaike information criteria
AOR: Adjusted Odds Ratio
BIC: Bayesian information criteria
EDHS: Ethiopian Demographic health survey
DIC: Deviance Information Criterion
ICC: Intra-Class Correlation
LLR: Log-Likelihood Ratio
MOR: Median Odds Ratio
PCV: Proportion of Variance Change
